# Blackening of titanium dioxide nanoparticles by atomic hydrogen and the effect of coexistence of water on the blackening

**DOI:** 10.1039/d0ra09090e

**Published:** 2021-01-21

**Authors:** Masahide Fujimoto, Masuaki Matsumoto, Naoki Nagatsuka, Katsuyuki Fukutani

**Affiliations:** Tokyo Gakugei Univ. 4-1-1 Nukui-kita-machi Koganei-shi Tokyo 184-8501 Japan masuaki@u-gakugei.ac.jp; Institute of Industrial Science, The University of Tokyo 4-6-1 Komaba Meguro-ku Tokyo 153-8505 Japan fukutani@iis.u-tokyo.ac.jp; Advanced Science Research Center, Japan Atomic Energy Agency Tokai Ibaraki 319-1195 Japan

## Abstract

A fast blackening process of titanium dioxide nanoparticles by exposing to atomic hydrogen was studied by estimating the color of the nanoparticles. The whiteness of TiO_2_ decreased exponentially with time, which suggests a first-order reaction between atomic H and surface oxygen, whose rate constant is proportional to the ambient pressure of H_2_. The rate constant increases as the temperature of nanoparticles at exposing to atomic hydrogen. The structure and size of nanoparticles were estimated by the X-ray diffraction (XRD), which shows that a part of anatase transferred to rutile and the crystal sizes of both anatase and rutile increased by hydrogenation above 600 K. The blackening of TiO_2_ halfway stopped under the condition of the similar partial pressure of water with hydrogen. This suggests the presence of reverse reaction between H_2_O and oxygen vacancy, whose reaction rate constant is proportional to the partial pressure of H_2_O.

## Abstract 2

A fast blackening process of titanium dioxide nanoparticles by exposing to atomic hydrogen was studied by estimating the color of the nanoparticles.

## Graphical abstract

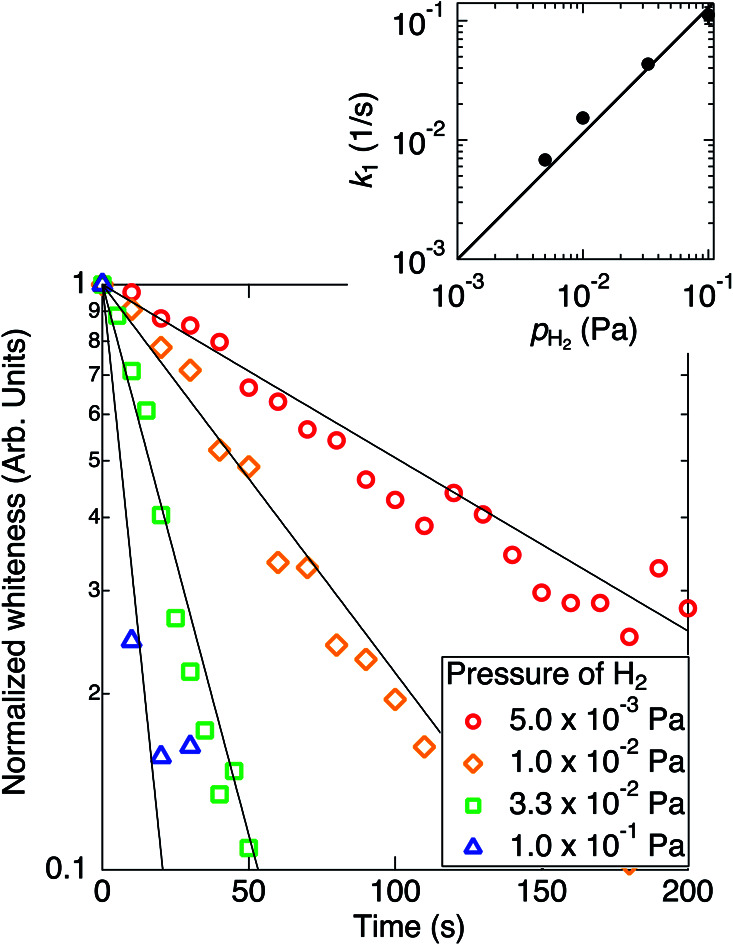

## 1 Introduction

Titanium dioxide (TiO_2_) is one of the most widely used photocatalysts and has been extensively studied after the pioneering work by Fujishima and Honda.^1,2^ Since the band-gap of TiO_2_ is wide (about 3 eV), it can absorb only ultraviolet light, which is contained only several % in the solar light. Therefore, many efforts have been made to improve the optical absorption of TiO_2_ under the solar light, such as generation of donor or acceptor states in the band gap by doping impurities^3–6^ or production of Ti^3+^ by self-doping.^7^ An alternative approach to improve absorption of solar light was proposed by Chen *et al.*,^8–10^ and reviewed by Liu *et al.*^11^ Chen *et al.* hydrogenated the anatase TiO_2_ nanoparticles in a 20 bar H_2_ atmosphere at about 473 K for 5 days. While the color of natural TiO_2_ nanoparticles is white, it changed to black by hydrogenation, therefore, it is called the black TiO_2_ (titania). The narrowing of the band gap was confirmed by X-ray photoelectron spectroscopy and the acceleration of photocatalytic decomposition of methylene blue was observed. Disordering of the outer region of nanoparticles was also observed by high-resolution transmission electron microscopy, which they attributed to the cause of the blackening and the band gap narrowing of TiO_2_. Selcuk *et al.* studied the formation mechanism and structure features of the black TiO_2_ using the first-principles-validated reactive force field molecular dynamics simulations and revealed that surface oxygen vacancies were created by reaction with H_2_ and its diffusion into bulk is inhibited by a high barrier in the subsurface region, which initiates surface disordering, and that this hydrogenated amorphous shell has a key role in the photoactivity of the black TiO_2_.^12^ The interaction of hydrogen with TiO_2_ has been extensively studied both experimentally and theoretically.^2^ The reduction of TiO_2_ and the production of oxygen vacancies or Ti^3+^, which modify the electronic structure, can be easily caused by vacuum annealing, hydrogen adsorption, electron irradiation, ultraviolet light irradiation, or exposure to molecular or atomic hydrogen.^13–16^ Among these procedures, the exposure to atomic hydrogen is expected to reduce the TiO_2_ surface effectively. In this paper, the blackening of the TiO_2_ nanoparticles by exposing to atomic hydrogen was studied at various sample temperatures and ambient H_2_ pressures. Since H_2_O is expected to be one of the products by the reduction of TiO_2_ by hydrogen and it was reported that the presence of water affects the stability of oxygens and oxygen vacancies at the surface and subsurface sites,^17^ the effect of partial pressure of H_2_O on the blackening was also studied.

## 2 Experimental

Blackening of TiO_2_ nanoparticles was performed in a high-vacuum chamber, whose base pressure was about 10^−4^ Pa. The anatase TiO_2_ nanoparticles with an average diameter of 8 nm (ST-01, Ishihara Sangyo Kaisha, Ltd.) were put on a W boat, which could be heated up to 873 K measured by the type K thermocouple. The temperature of the W boat is called the hydrogenation temperature below. The atomic hydrogen was produced by a W filament that was placed about 5 mm from the boat. The filament was heated at about 1973 K by direct current, of which temperature was monitored by a pyrometer. It was reported that the cracking efficiency of H_2_ was 1.5% at 1873 K and increases exponentially with W filament temperature, which suggests that the atomic hydrogen can be efficiently produced at 1973 K.^18^

After setting the temperature of the W boat and filament at appropriate temperatures, photos of the sample were taken every several seconds. The whiteness of the sample was estimated from the numerical value of the 8 bit digitized monochrome image by using an imaging software. After introducing H_2_ into vacuum chamber, time evolution of the whiteness was obtained. The whiteness was normalized by the initial value of a series of the measurements. Since only the part of the nanoparticles facing to the W filament was blackened after a measurement of time evolution, the whiteness could be initialized by scrambling with a wobble stick and the next measurement was started. The speed of blackening largely depends on the distance between the sample and the W filament for H_2_ dissociation. It is necessary to put the W filament near the nanoparticles to blacken them quickly. Though the distance affects the temperature of the sample, this effect is included in the increase of the hydrogenation temperature. It is therefore suggested that the efficient incidence of atomic hydrogen is important for the effective blackening of TiO_2_.

Structural phase and average size of the nanoparticles were estimated by X-ray diffraction (XRD: Ultima IV, RIGAKU). The fraction of anatase was estimated by the integrated peak intensities for the anatase and rutile. The average size (*D*) of the nanoparticles was estimated by the Scherrer equation
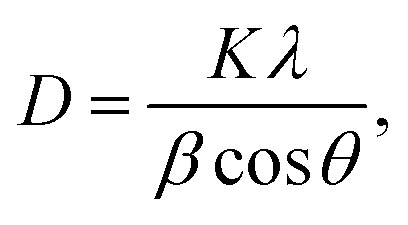
where *β* was the full-width at half-maximum (FWHM) obtained by the Lorentzian-fitted diffraction peak, *θ* is the angle of the peak of XRD, *λ* = 0.1541 nm is the wavelength of Cu Kα and *K* = 0.9 is the form factor.

## 3 Results

**Fig. 1:**
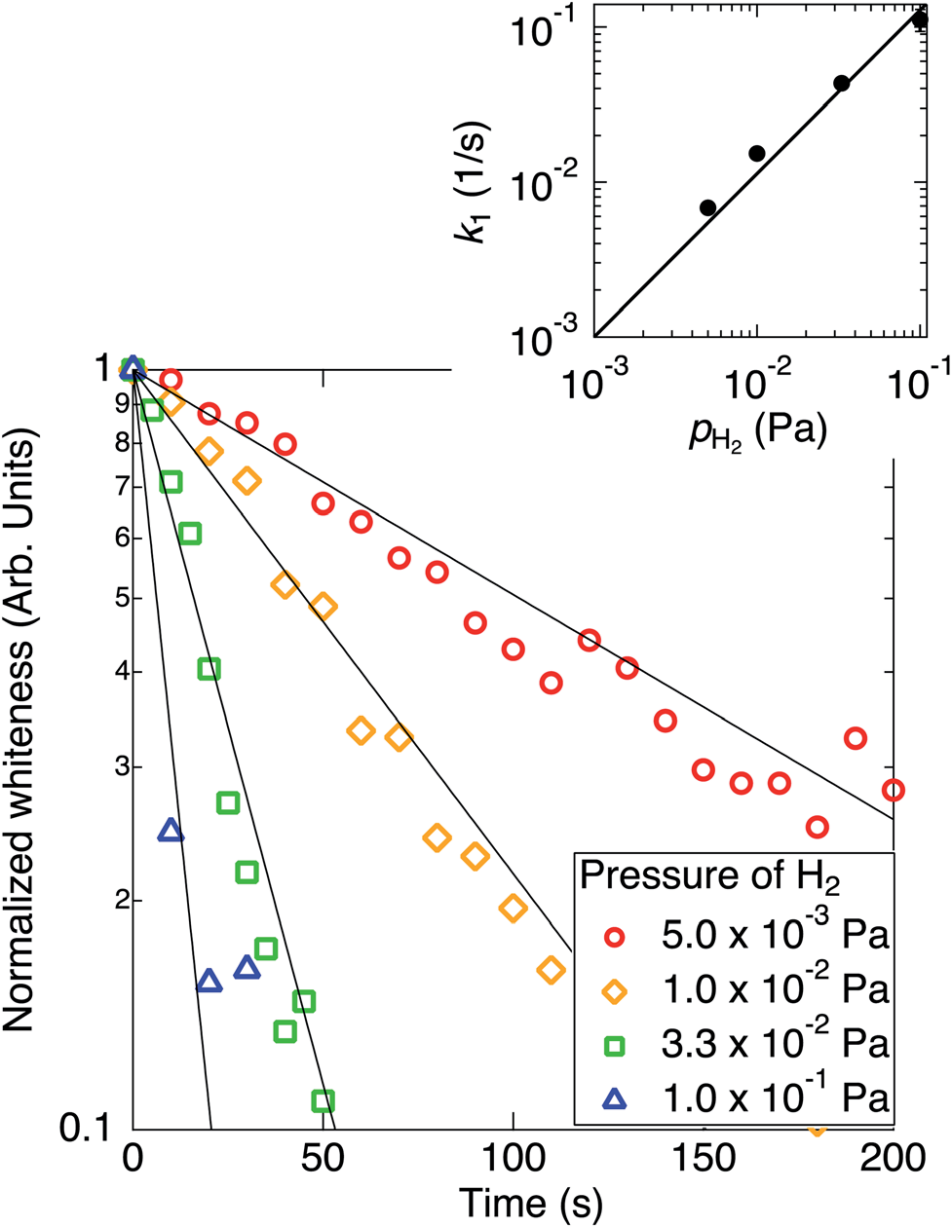
Semi-logarithmic plot of the time evolutions of the normalized whiteness of TiO_2_ nanoparticles at various ambient pressures of H_2_ from 5.0 × 10^−3^ Pa to 1.0 × 10^−1^ Pa. The temperatures of nanoparticles and W filament were kept at 573 K and 1973 K, respectively. (Inset) Double logarithmic plot of the inverse values of initial time constants against the H_2_ pressures.

The temperatures of TiO_2_ nanoparticles and W filament were kept at 573 K and 1973 K, respectively, for about 30 min to get the stable states, and H_2_ was introduced in vacuum at *t* = 0. When the ambient H_2_ pressure (*p*_H_2__) was kept constant at a fixed pressure from 5.0 × 10^−3^ Pa to 1.0 × 10^−1^ Pa, the color of the nanoparticles changed from white to black and sintering occurred in a few minutes. The particle size increased by sintering. When the sample was treated without introducing H_2_ (at the base pressure of 10^−4^ Pa), only the sintering occurred but the color of the nanoparticles hardly changed. Time evolutions of the whiteness of TiO_2_ nanoparticles at various *p*_H_2__ are shown in Fig. 1. As shown in the figure, the whiteness decreased almost exponentially with time and the time constant became smaller as the ambient H_2_ pressure was increased.

**Fig. 2:**
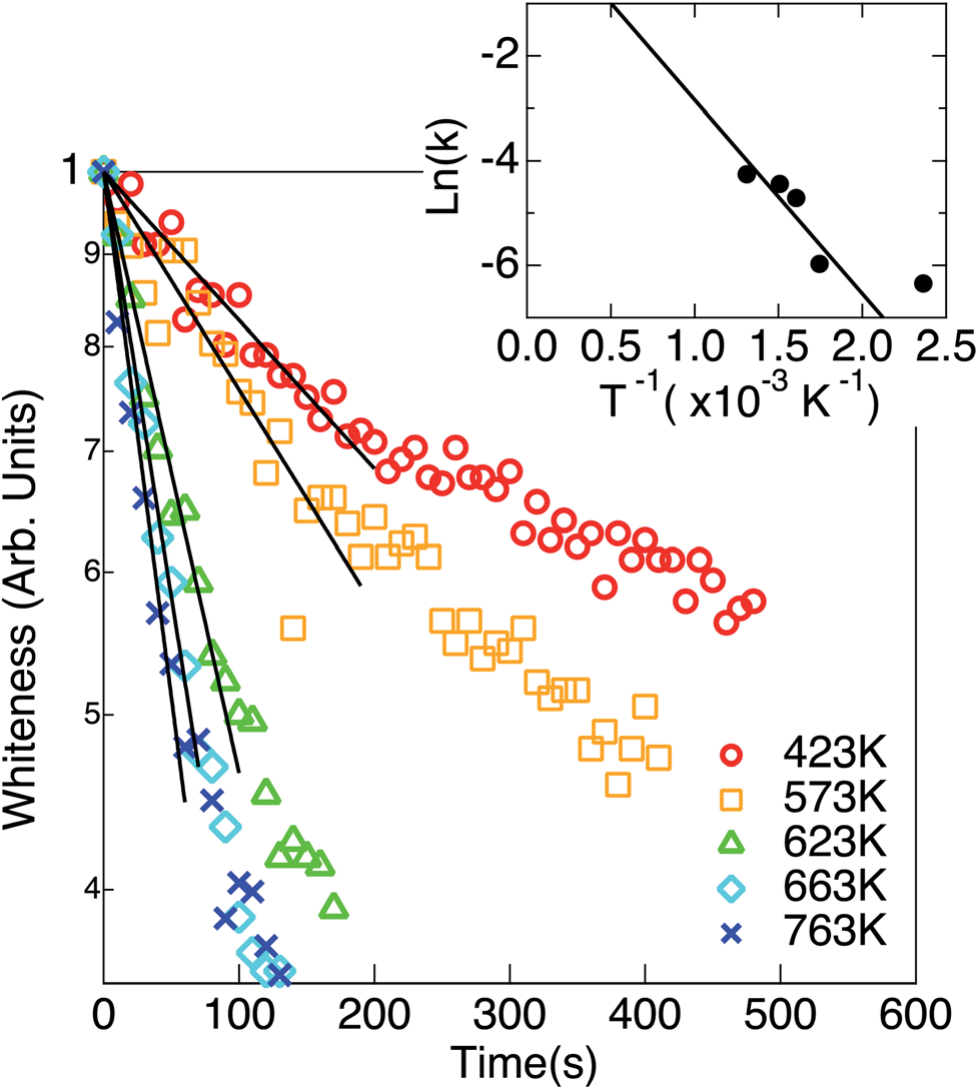
Semi-logarithmic plot of the time evolutions of the normalized whiteness of TiO_2_ nanoparticles at various hydrogenation temperatures from 423 K to 763 K. The temperature W filament was kept at 1973 K and the ambient H_2_ pressure was kept at 1.0 × 10^−2^ Pa. The exponentially fitted curves using initial evolutions are plotted by the solid lines. (Inset) Semi-logarithmic plot of the inverse values of initial time constants against the inverse of temperature.

Time evolutions of the whiteness of TiO_2_ nanoparticles at various hydrogenation temperatures (*T*_s_) are shown in Fig. 2. The whiteness initially decreased exponentially, and the initial time constants *τ*_1_ decreased as the hydrogenation temperature increased. Decreasing curves of the whiteness deviate from the exponential functions at the low whiteness region.

Though the TiO_2_ nanoparticles could be blackened in a few minutes under the low ambient H_2_ pressures, the blackening only occurs at the upper side of the nanoparticles facing to the filament for H_2_ dissociation, which means that the atomic hydrogen is indispensable for the blackening of TiO_2_. It is necessary to scramble the nanoparticles every several minutes by the wobble stick to get the uniform black nanoparticles for the XRD experiments.

**Fig. 3:**
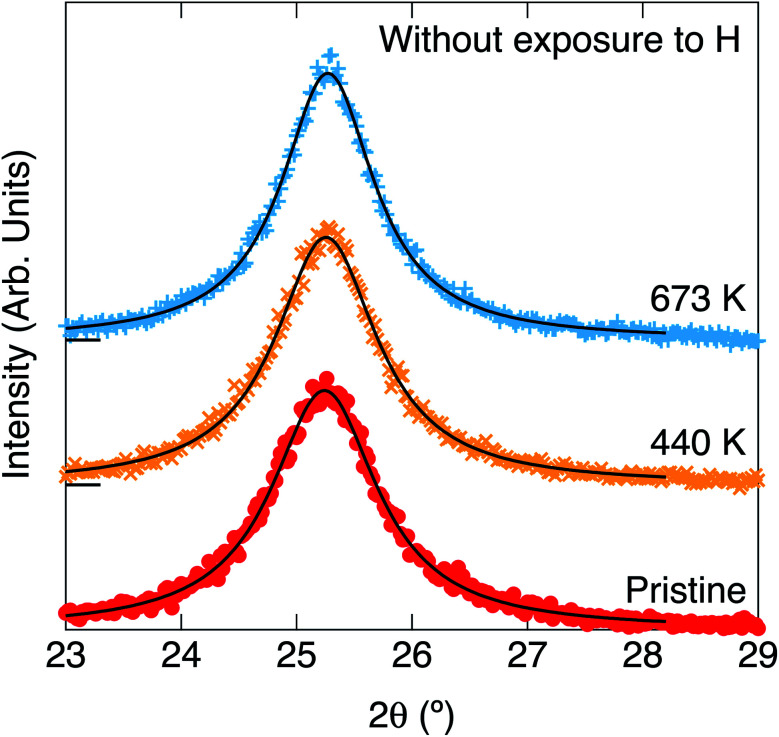
X-ray diffraction spectra of the pristine white anatase nanoparticles and after annealing at 440 K and 673 K without exposure to hydrogen. The blackening did not occur. The peaks at 2*θ* = 25.2° is from anatase (101). The Lorentzian fitted curves were drawn by the solid lines.

**Fig. 4:**
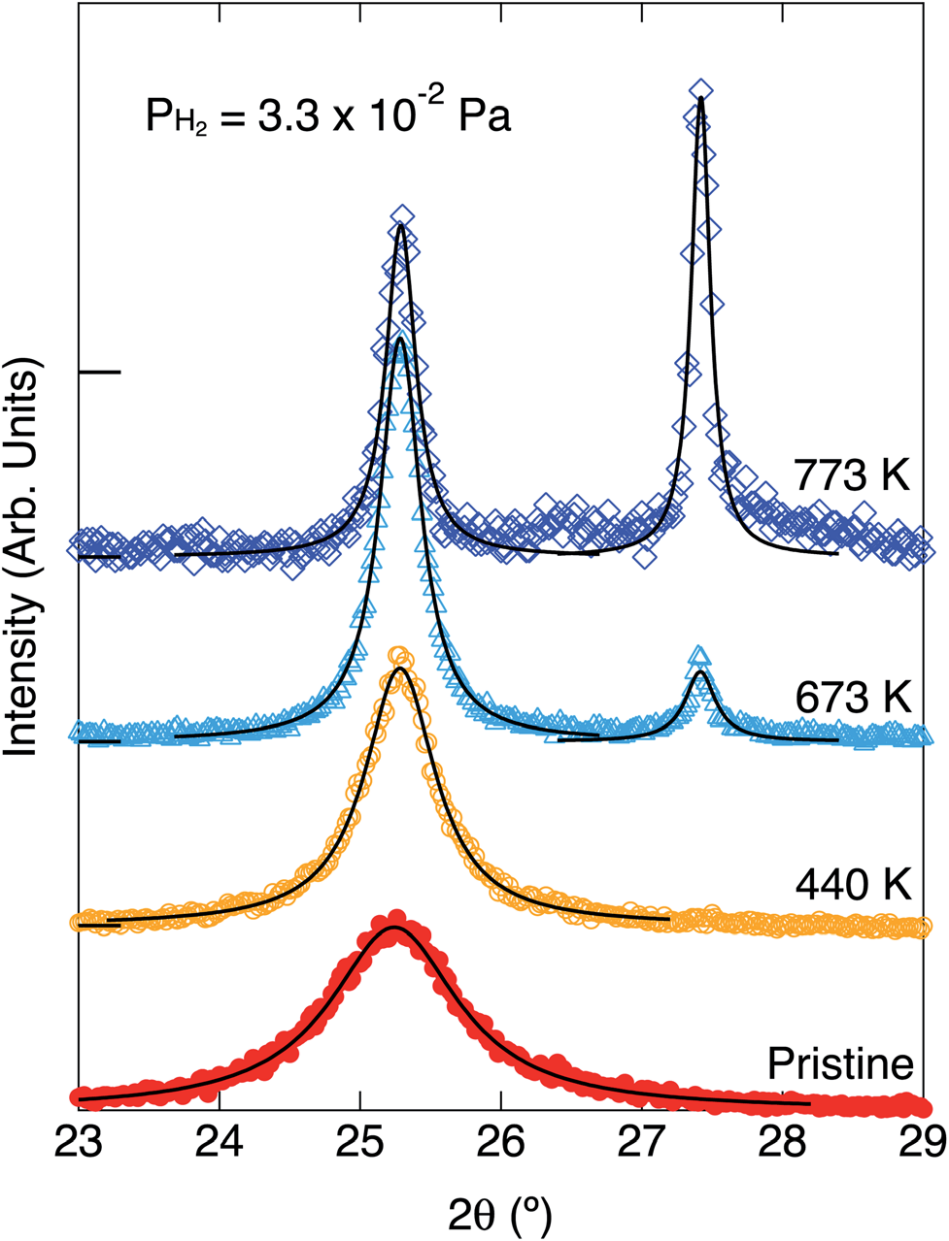
X-ray diffraction spectra of the pristine white anatase nanoparticles and the black TiO_2_ produced at 440 K, 573 K, 673 K and 773 K. The peaks at 2*θ* = 25.2° and 2*θ* = 27.5° are from anatase (101) and rutile (110), respectively. The Lorentzian fitted curves were drawn by the solid lines.

XRDs for the pristine white TiO_2_ and the heated ones without and with exposure to hydrogen are shown in Fig. 3 and 4, respectively. The parameters of the XRD are summarized in Table 1. The TiO_2_ nanoparticles were not blackened without exposure to hydrogen. Only one peak can be observed at 25.3° for the pristine white TiO_2_ nanoparticles. The peak position did not change by heating the white TiO_2_ nanoparticles without exposure to hydrogen. The peak width slightly narrowed by heating, which means that the average crystal size slightly increased from 7.32 nm to 8.36 nm.

**Table 1:** Table of the color (black or white) and the crystal structure (anatase or rutile) of TiO_2_, the presence of hydrogen at heating (yes/no), the sample temperature at heating or hydrogenation (*T*_s_), 2*θ* at the peak, the width of the peak, the fraction of anatase, the size of nanoparticles are shown

Color (B/W)	Anatase/rutile	H exposure	*T*_s_ (K)	Peak 2*θ* (°)	Peak width (°)	Fraction	Crystal size (nm)
W	A	n	—	25.2	1.112	1	7.32
W	A	n	440	25.3	1.073	1	7.59
W	A	n	673	25.3	0.9745	1	8.36
B	A	y	440	25.3	0.6104	1	13.3
B	A	y	673	25.3	0.3552	0.89	22.9
B	R	y	673	27.4	0.2593	0.11	31.5
B	A	y	773	25.3	0.2575	0.53	31.6
B	R	y	773	27.4	0.1647	0.47	49.7

**Fig. 5:**
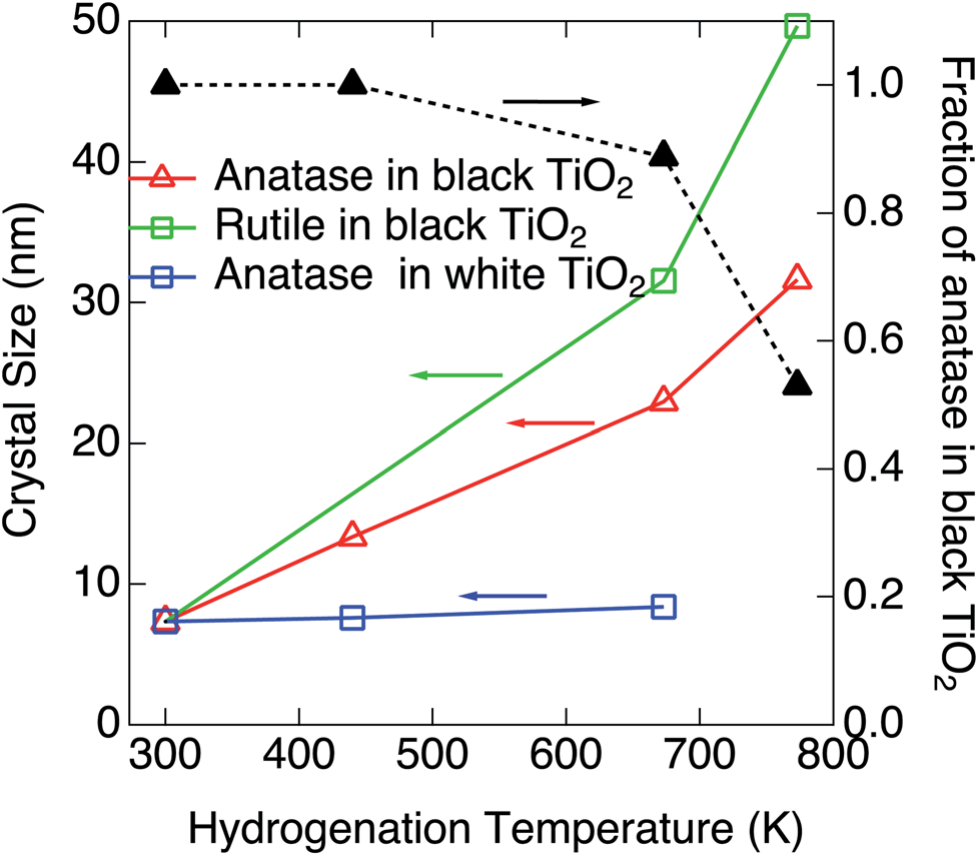
Hydrogenation temperature dependences of the fraction of anatase and the size of nanoparticles and crystal structure at a fixed ambient H_2_ pressure of 3.3 × 10^−2^ Pa.

Blackening of TiO_2_ was caused by heating under exposure to the atomic hydrogen. XRDs for the black TiO_2_ produced at the hydrogenation temperatures of 440 K, 673 K and 773 K are shown in Fig. 4. XRD for the pristine white TiO_2_ is also shown for comparison. The width of the peak at 25.3° narrowed by heating, which suggests that the average crystal size increased from 7.32 nm to 13.3 nm, 22.9 nm and 31.6 nm by heating at 440 K, 673 K and 773 K, respectively. Besides the change in the peak width, another new peak appeared at 27.4° after blackening at and above 673 K. The peak at 25.3° and 27.4° could be assigned to the anatase (101) and rutile (110), respectively, which means that a part of the original anatase TiO_2_ transferred to rutile at high hydrogenation temperatures. The fractions of anatase were estimated by the integrated intensities of the two peaks. Temperature dependences of the fraction of anatase and the crystal sizes are shown in Fig. 5. They are also summarized in Table 1. As the hydrogenation temperature was increased, the fraction of anatase started to decrease above 500–600 K, from 100% at 440 K to 50% at 780 K. The crystal sizes also increased as the hydrogenation temperature increased. This result is consistent with the previous reports by Mao *et al.*, which showed that the phase transition from anatase to rutile is accelerated by H_2_ annealing.^19^

**Fig. 6:**
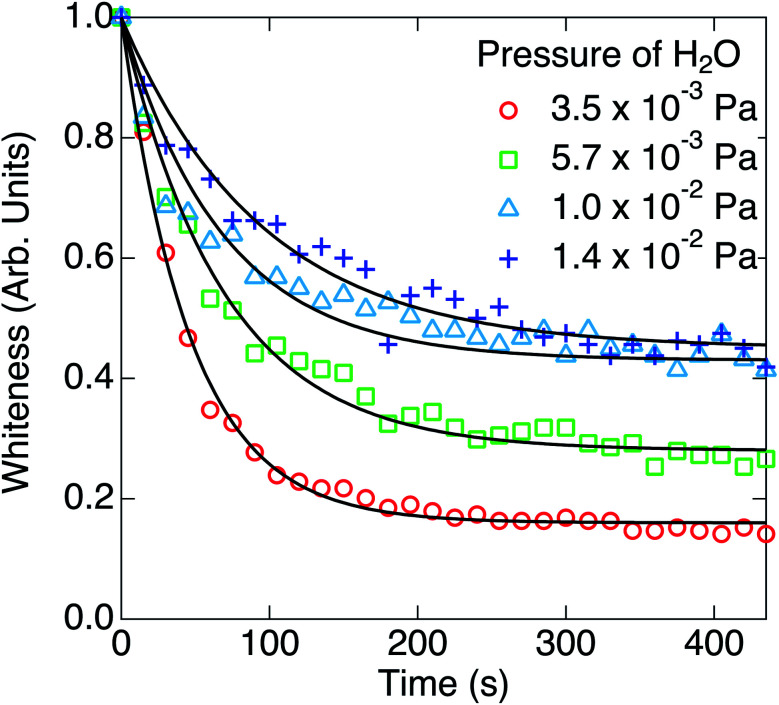
Time evolutions of the whiteness at the partial pressures of H_2_O of 3.5 × 10^−3^ Pa, 5.7 × 10^−3^ Pa, 1.0 × 10^−2^ Pa and 1.4 × 10^−2^ Pa. The ambient partial pressure of H_2_ was 3.3 × 10^−2^ Pa. The solid lines are exponentially fitted curves.

Finally, the effect of coexistence of water impurity was researched. Time evolutions of the whiteness at the partial pressures of H_2_O (*p*_H_2_O_) of 3.5 × 10^−3^ Pa, 5.7 × 10^−3^ Pa, 1.0 × 10^−2^ Pa and 1.4 × 10^−2^ Pa are shown in Fig. 6. The base pressure before introducing water was 5.0 × 10^−5^ Pa. After setting the partial pressures of H_2_O to respective values, the temperatures of nanoparticles and W filament were kept at 573 K and 1973 K, respectively, for about 30 min, and H_2_ with a partial pressure of 3.3 × 10^−2^ Pa was introduced into the chamber at *t* = 0 s. As shown in Fig. 6, the whiteness decreased exponentially, but they saturated to the larger values than those in Fig. 1, which means that the blackening of TiO_2_ nanoparticles halfway stopped.

## 4 Discussion

It is thought that the disordering of the outer region of the nanoparticles is related to the blackening from the results by HRTEM.^8^ This disordering is thought to be caused by the reduction of TiO_2_, which produces oxygen vacancies and/or interstitial hydrogen near the surface. Since the production of OH does not cause blackening^16^ and the H amount is confirmed to be smaller than 1 atomic% by nuclear reaction analysis,^21^ the production of oxygen vacancies is focused and discussed in this manuscript.

The reaction between the atomic hydrogen from the high temperature W filament and the surface oxygen (O_s_) of TiO_2_ nanoparticles can be written by the following formula.^11,12^

1 2H + O_s_ → H_2_O + V_O_

A H_2_O molecule and a surface oxygen vacancy (V_O_) are produced by the reaction. One possible assumption is that the nanoparticle turns black as the number of V_O_ increases and that of O_s_ decreases. In the following discussion, therefore, it is assumed that the whiteness of the nanoparticle is proportional to the coverage of the surface oxygen O_s_.

If the number of H_2_ is enough and the Arrhenius type rate equation is assumed in this reaction, the coverage of surface oxygen ([O_s_]) obeys the following differential equation.2
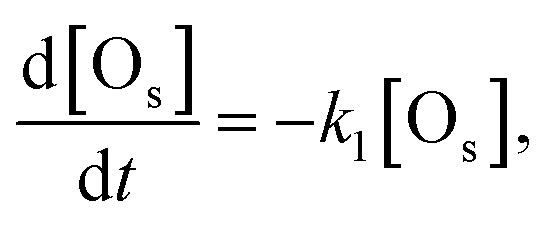
where *k*_1_ is the reaction rate constant. Assuming that the whiteness is proportional to [O_s_], the time evolution of the whiteness (*W*) can be written as follows,

3 *W* ∝ [O_s_] ∝ exp(−*k*_1_*t*),

4 *k*_1_ = *A*_1_ exp[−*E*_a_/(*k*_B_*T*_s_)],

where *A*_1_ is the frequency factor, *E*_a_ is the activation energy for the reaction (1), *T*_s_ is the temperature of the surface of TiO_2_, and *k*_B_ is the Boltzman constant. The reaction rate constants (*k*_1_) at the initial stages of the reaction are plotted against *p*_H_2__ in the inset of Fig. 1, which shows their proportionality. Since *T*_s_ is same for all curves in Fig. 1, *k*_1_ is proportional to the frequency factor *A*_1_. At the initial stage of the reaction, the number of O_s_ is enough, it is therefore suggested that the frequency factor depends on the number of incident H atoms. Since the incident frequency (*Γ*) of H atoms is proportional to 
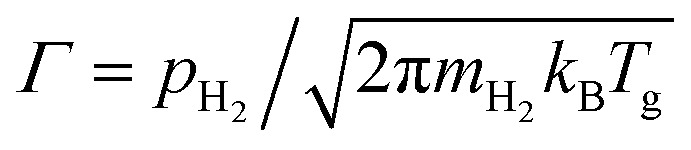
, where *m*_H_2__ and *T*_g_ are the mass and the temperature of ambient H_2_ molecules, it is reasonable that the reaction rate constant is proportional to the ambient pressure of H_2_.

In Fig. 2, the whitenesses also change exponentially at the initial stages of the reaction. However, it is difficult to fit each curve by a single rate constant. The slopes of the curves significantly change in the blackening process. This suggests a possibility of the presence of two blackening processes with different reaction rate constant, although this cause is not known yet.

It should be noted that the absolute value of the reaction constant at 573 K in Fig. 2 is different from that at 1.0 × 10^−2^ Pa in Fig. 1 in spite of the similar condition of reaction. As described in the Experimental section, this is because the distance between the sample and the W filament is different from each other. Though the absolute value of the rate constant cannot be discussed, the temperature dependence of the relative rate constant is discussed.

The Arrhenius plot for the reaction (eqn (4)) is plotted in the inset of Fig. 2. The activation energy for the reaction (1) was estimated to be 186 ± 50 meV. Scheiber *et al.* reported that migration of V_O_ from surface to subsurface starts at temperatures ∼200 K, which suggests that diffusion of O from subsurface to surface also starts at such low temperatures.^20^ Therefore, it is suggested that the diffusion of V_O_ or O are not a rate-determining process for the reaction (1). Ohashi *et al.* reported that the tailing feature at the valence band maximum that is characteristic to the black TiO_2_ was not observed by annealing after H ion irradiation, where no structural disorder occurred by H incorporation in TiO_2_ without annealing.^16^ In the present work, on the other hand, the TiO_2_ could be blackened by annealing under the atomic H irradiation.

Since H_2_O is the product of the reaction (1), the reverse reaction also occurs if a large amount of H_2_O gas exists in the atmosphere.

5 H_2_O + V_O_ → H_2_ + O_s_

If the number of incident H_2_O gas is enough and the first order rate equation is also assumed in this reaction, the differential equation for the two reactions (1) and (5) can be written as follows.6
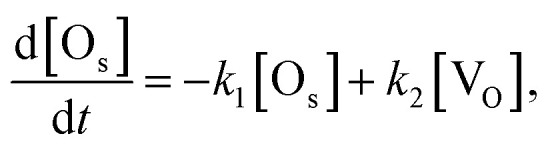
7
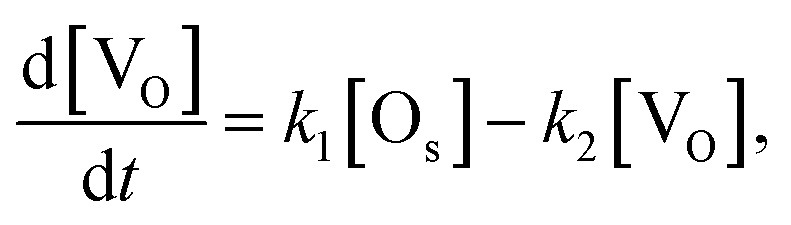
where [O_s_] and [V_O_] are the coverages of surface oxygen and oxygen vacancy, respectively, which fulfils [O_s_] + [V_O_] = 1. *k*_1_ and *k*_2_ are the reaction rate constants of the two reactions (1) and (5), respectively. The solution of these differential equations is8
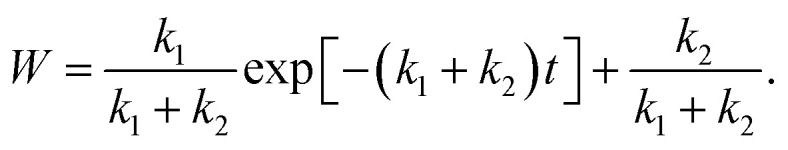
This solution is consistent with the trend of the exponential decay and the saturation of the whiteness in Fig. 6.

**Fig. 7:**
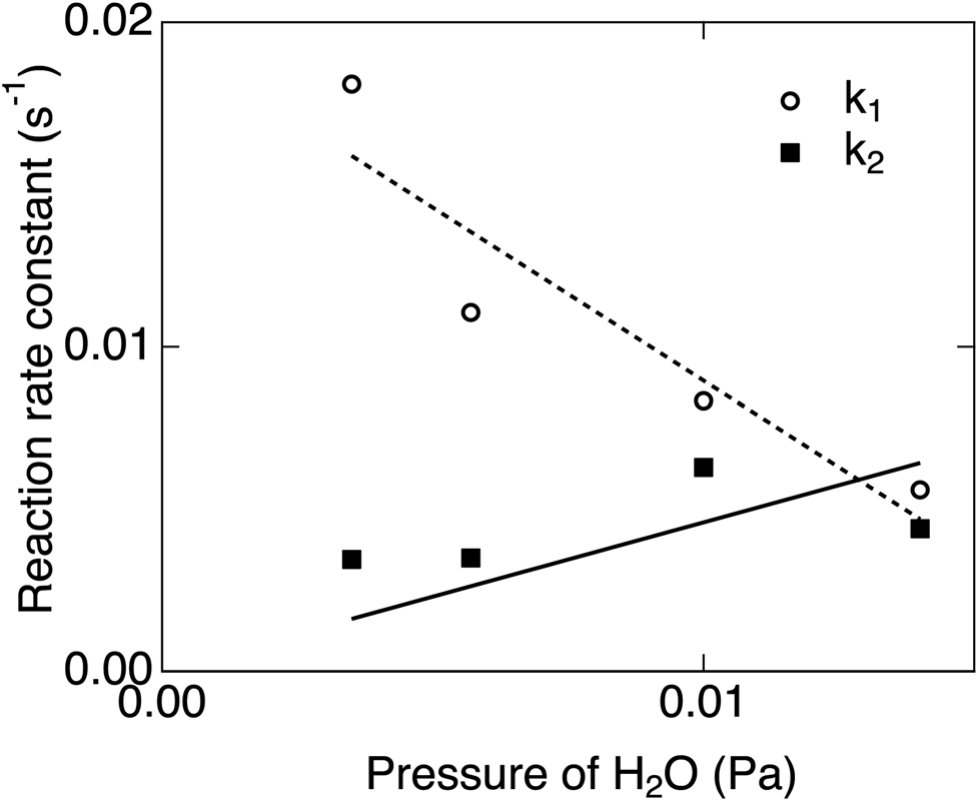
*p*_H_2_O_ dependences of *k*_1_ and *k*_2_ estimated from the rate constants and the saturation values of the whiteness in Fig. 6.

The parameters *k*_1_ and *k*_2_ were estimated from the reaction rate constants (*k*′ = *k*_1_ + *k*_2_) and the saturation values of the whiteness (*w*_s_ = *k*_2_/(*k*_1_ + *k*_2_)) by fitting the experimental curves to eqn (8) in Fig. 6. They are summarized in Table 2 and plotted in Fig. 7. Though the trends of increasing of *k*_2_ with increasing *p*_H_2_O_ are shown, the proportionality between *k*_2_ and *p*_H_2_O_, which is expected under the assumption of a similar equation with eqn (4), is not clear. It is also suggested that the reaction (1) between H_2_ and O_s_ is severely blocked by the presence of H_2_O, by which *k*_1_ largely decreases with increasing *p*_H_2_O_. Li and Gao reported that the surface V_O_ becomes more stable than the subsurface V_O_ by water adsorption, by which water dissociation can be activated. This may also increase *k*_2_, which is consistent with our results in Fig. 7.

**Table 2:** The reaction rate constants (*k*′), the saturation values (*w*_s_) of the time evolution of the whiteness at several partial pressures of H_2_O, *k*_1_ and *k*_2_ estimated by the results in Fig. 6

*p*_H_2_O_/Pa	*k*′/s^−1^	*w*_s_	*k*_1_/s^−1^	*k*_2_/s^−1^
3.50 × 10^−3^	0.0215	0.16	0.018	0.003
5.70 × 10^−3^	0.0146	0.28	0.011	0.003
1.0 × 10^−2^	0.0146	0.43	0.008	0.006
1.40 × 10^−2^	0.0100	0.44	0.006	0.004

## 5 Summary

The blackening process of TiO_2_ nanoparticles by exposing to atomic hydrogen was studied. The whiteness of TiO_2_ decreased exponentially with time. Assuming that the whiteness is proportional to the coverage of the surface oxygen vacancy, the rate constant in the first order rate equation for the reaction between H_2_ and surface oxygen is proportional to the pressure (the impinging rate) of H_2_, and increases with increasing the hydrogenation temperature. The crystal size and the anatase/rutile ratio were estimated by XRD, which shows that the decrease in the fraction of anatase begins almost at the same hydrogenation temperature with the increase in the size of TiO_2_ nanoparticles. The blackening of TiO_2_ halfway stopped under the condition of the similar partial pressure of water with hydrogen. This suggests the presence of reverse reaction between H_2_O and oxygen vacancy and the presence of H_2_O severely blocks the reaction between H_2_ and surface oxygen. Tailing of the valence band maximum and the enhancement of photocatalytic reaction were confirmed for the single crystal TiO_2_ blackened by the same method, and the surface structure is under study.^21^ It is expected that our new method would contribute the efficiency improvement of photocatalyst based on TiO_2_.

## Conflicts of interest

There are no conflicts to declare.

## Supplementary Material
